# Quantifying changes in triaxial seismocardiography variability due to sub-optimal volume status

**DOI:** 10.3389/fphys.2026.1823345

**Published:** 2026-05-18

**Authors:** Gabriela I. Cestero, Zeineb Bouzid, Afra Nawar, Cem O. Yaldiz, Demet Tangolar, Zhongjun J. Wu, Jin-Oh Hahn, Omer T. Inan

**Affiliations:** 1 School of Electrical and Computer Engineering, Georgia Institute of Technology, Atlanta, GA, United States; 2 Department of Surgery, University of Maryland School of Medicine, Baltimore, MD, United States; 3 Department of Mechanical Engineering, University of Maryland, College Park, MD, United States

**Keywords:** blood volume status, hypervolemia, hypovolemia, lateral SCG axis, seismocardiogram (SCG), signal consistency, signal variability, triaxial SCG

## Abstract

**Introduction:**

Hypervolemia (volume overload) and hypovolemia (volume deficit) are sub-optimal blood volume conditions that influence overall circulation and organ function, with substantial shifts posing serious risk of death. In both conditions, the body’s response mechanisms can compensate for sub-optimal volume up to a point at which the cardiovascular system decompensates. Most current methods for assessing blood volume status are either limited to clinical settings or are inaccurate, posing a particular challenge for triaging or monitoring in austere environments. Recent work has aimed at developing novel wearable sensing and machine learning technologies to yield early and accurate signs of sub-optimal volume status prior to decompensation. Seismocardiogram (SCG) signals, representing vibrations of the chest wall due to heartbeat, have been leveraged as a non-invasive and convenient means of extracting cardiac timing representative of sub-optimal volume status. In this work, we aimed to elucidate the changes in morphological variability in the SCG signal in both hypervolemic and hypovolemic conditions. We hypothesized that the variability in the SCG signal would be heightened in decompensated versus compensated states, since the decompensated state would represent hemodynamic conditions that were less stable and thus more variable on a beat-by-beat basis.

**Methods:**

Two datasets were fused for this analysis: 11 heart failure patients with acute decompensation and hypervolemia undergoing vasodilation (thus transitioning to a compensated state), and 15 swine undergoing a volume depletion experiment (hypovolemia). SCG signal consistency, representing the inverse of signal variability, was computed using 60 seconds of data extracted each from the compensation and decompensation periods.

**Results:**

While there was no significant change in signal consistency from compensation to decompensation in the dorso-ventral nor head-to-foot SCG axes for either the hypervolemia or hypovolemia populations, we observed a significant change in signal consistency from compensation to decompensation in the lateral axis for both the hypervolemia (p = 0.042) and hypovolemia (p = 0.012) populations.

**Discussion:**

These results demonstrate an important relationship between compensation status and SCG morphology variability, particularly in the underutilized SCG lateral axis. This paper thus sets a foundation for enabling future algorithms leveraging SCG variability to provide baseline-free estimation capability of sub-optimal volume status in field settings.

## 1 Introduction

The performance and health of the heart with each beat hinges on its being filled with the proper volume of blood: too much filling can lead to volume overload, where the ventricular muscles experience excessive strain and weaken with time (Melenovsky, 2013); too little filling results in volume deficit, where the heart’s insufficient filling leads to weak and low pulsatile output, and thereby poor perfusion at the tissues and organs (Kreimeier, 2000). Volume overload, also called hypervolemia, is commonly associated with heart failure (Ghali and Tam, 2010) and kidney disease (Ekinci et al., 2018), but it can also occur as a form of heat adaptation (Convertino et al., 1980), during long-term exposure to high altitudes (Siebenmann et al., 2017), or from overhydration after extreme exercise (Montain et al., 2001). Volume deficit, also called hypovolemia, can be produced by two mechanisms. Absolute hypovolemia is caused by intravascular fluid loss and is commonly associated with trauma and hemorrhage (Gutierrez et al., 2004), but it can also occur in extreme environments due to dehydration (Barrow and Clark, 1998; Kreimeier, 2000) or initial exposure to high altitudes (Siebenmann et al., 2013). Relative hypovolemia is caused by increased vascular capacitance and is commonly associated with sepsis (Perner et al., 2018) but can also occur with heat stress (Barrow and Clark, 1998). Both hypervolemia and hypovolemia can lead to poor tissue perfusion, organ failure, and ultimately mortality (Kreimeier, 2000; Vincent, 2019).

In sub-optimal volume status, the normal compensatory mechanisms of the body can account for the condition until a point when the person “decompensates” (Segizbaeva et al., 2015; Miller, 2016; Suresh et al., 2019). Accordingly, the clinical status of patients experiencing both hypervolemia or hypovolemia are typically considered either “compensated” or “decompensated”, usually defined based on physiological measurements. Measurements assessing blood volume status include invasive catheter-based central venous pressure and pulmonary capillary wedge pressure (PCWP), blood biomarkers, microcirculatory perfusion parameters, indicator-dilution blood volume analysis, chest radiography, and ultrasound of the heart, lungs, and large blood vessels (Kalantari et al., 2013; Elhassan et al., 2021; Koratala et al., 2022). However, these measurements are expensive and limited to clinical settings only. Conversely, conventional vital signs such as heart rate, heart rate variability (HRV), and non-invasive blood pressure are easy to measure, but they are non-specific and typically late markers of worsening state towards decompensation (Androne et al., 2004; Frank Peacock and Soto, 2010; Convertino et al., 2013; Elhassan et al., 2021).

Recently, researchers have evaluated non-invasive wearable sensor-based approaches to assessing volume status without the need for invasive hemodynamic measurements and with better sensitivity as compared to conventional vital signs. Approaches for monitoring hypovolemia include a globalized machine learning model utilizing electrocardiogram (ECG), seismocardiogram (SCG), and photoplethysmogram (PPG) signal-derived amplitude and temporal features (Zia et al., 2020b; Kimball et al., 2021), as well as the PPG finger-cuff-based compensatory reserve measurement (Roden et al., 2024). Approaches for monitoring hypervolemia include artificial intelligence estimation of PCWP using ECG, SCG, and PPG signals collected from the CardioTag device (Cardiosense, Inc., Chicago, Illinois, USA) (Klein et al., 2025) and the ZOLL Heart Failure Management System (ZOLL Medical Corporation, Chelmsford, Massachusetts, USA), used for clinician monitoring, which collects ECG, chest radiofrequency, and accelerometry data (Boehmer et al., 2024). Some of the highest performing methods among these leverage SCG signals, but the understanding of how these cardiogenic vibrations are affected by sub-optimal volume status is lacking in the existing literature. Studies aimed at providing better fundamental understanding of the characteristics of SCG signals that are affected by sub-optimal volume status, and the axes of the tri-axial SCG signals captured at the chest that are most informative for assessing volume status, can provide great value in both elucidating current approaches and delivering future opportunities for improved approaches.

**Figure 1:**
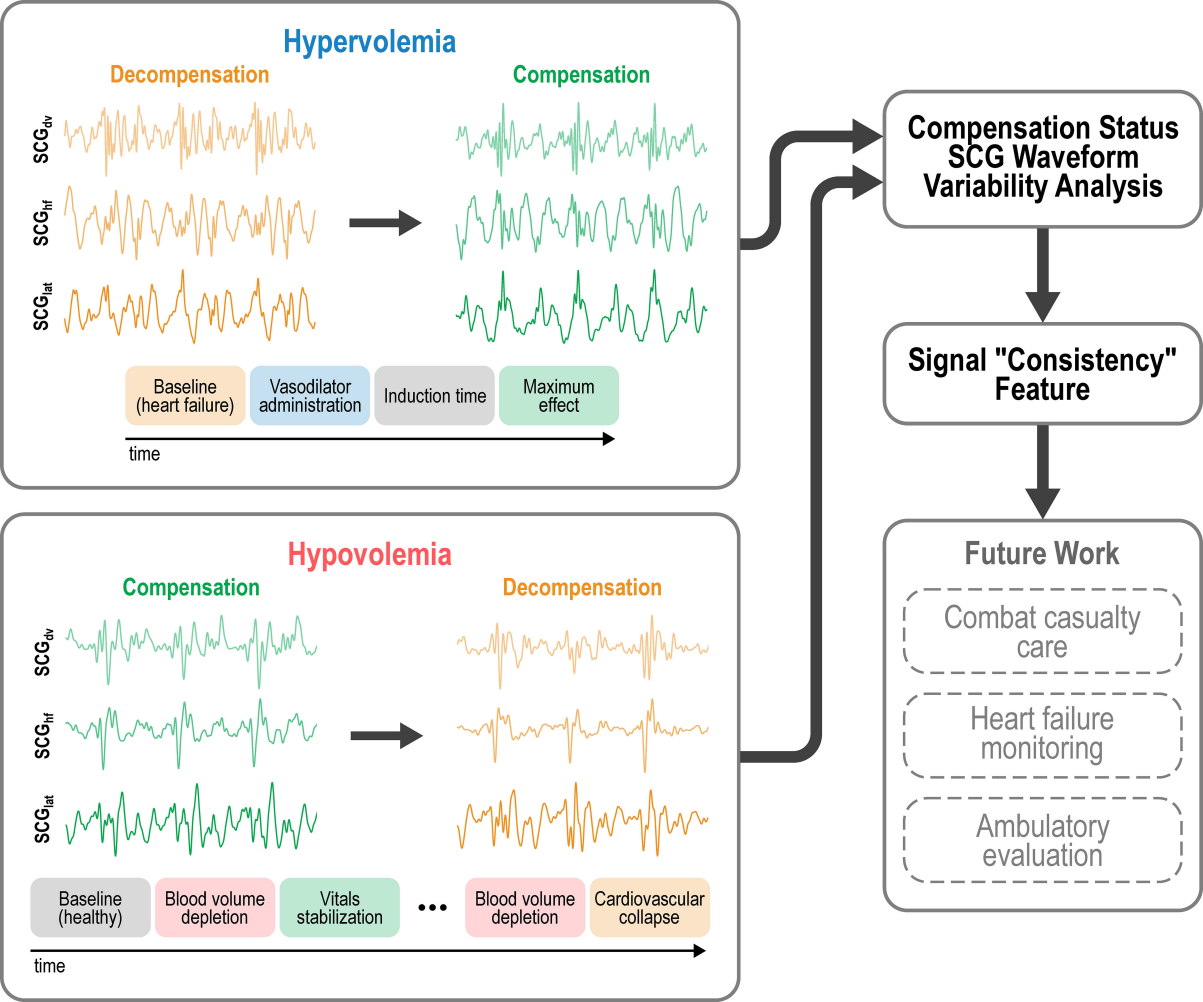
The overall framework proposing SCG variability analysis for various applications. Example SCG signals recorded in 3 axes (dv, dorso-ventral; hf, head-to-foot; lat, lateral) are shown.

In this work, we investigated the three-dimensional (triaxial) SCG signal, a measurement that can be captured readily in various settings using a simple accelerometer or medical devices such as the CardioTag and specifically examined the variability in triaxial SCG signals in sub-optimal volume status conditions. To generalize beyond any one condition or disease, we have examined both hypervolemia, via volume overload in human subjects with heart failure, and absolute hypovolemia, via volume depletion in a large animal model, and quantified SCG variability in both compensated and decompensated clinical states in both groups. This methodology is visualized in **Figure 1**. In the interest of conciseness, we will refer to absolute hypovolemia as simply “hypovolemia” for the remainder of this paper. Our assumption was that both cases of sub-optimal volume status would result in shared disturbances to the morphological characteristics of the SCG, and particularly that the variability of SCG signal shape throughout the recording would be demonstrably and significantly affected.

In this paper, our contributions are: (1) investigation of tri-axial SCG – not limited to the dorso-ventral SCG axis – under hypervolemia and hypovolemia conditions; (2) novel application of the signal consistency metric defined by Pandia (2017) to the SCG signal; (3) comparison of signal consistency between compensated and decompensated states of sub-optimal blood volume; and (4) the further support of the importance of lateral SCG in the differentiation between compensation and decompensation for both hypervolemia and hypovolemia. This is the first study to our knowledge that considers the impacts of both hypervolemia and hypovolemia on a single metric of the SCG and relates their physiological change from compensation to decompensation. The knowledge gained from this study could inform the development of a single wearable device capable of monitoring severity changes in both hypervolemia and hypovolemia, allowing remote, preventative evaluation of these conditions.

## 2 Materials and methods

To investigate the effects of compensatory state in the context of volume status on SCG waveform variability, 11 heart failure patients undergoing vasodilation, representing hypervolemia, and 15 swine undergoing hemorrhage, representing hypovolemia, were studied. ECG and SCG signals from 60 seconds during the compensated and decompensated states were extracted from the datasets. A period of 60 seconds was chosen as the long-term goal for this research is to develop a system that can quickly detect changes in blood volume status to enable rapid intervention.

ECG was used to segment SCG into heartbeats, and after removal of beats with arrhythmia or motion artifacts, a single SCG signal consistency value was computed for the compensated state and another for the decompensated state for each subject. Finally, a paired statistical test, the non-parametric Wilcoxon signed-rank test, was used to determine if signal consistency significantly changed from compensation to decompensation for the hypervolemia and hypovolemia populations.

### 2.1 Hypervolemia dataset

To study the effect of compensated versus decompensated hypervolemia on SCG, we utilized a publicly available dataset of heart failure patients undergoing right heart catheterization while wearing a wearable device to capture SCG and ECG signals (Goldberger et al., 2000; Shandhi et al., 2022; Chan et al., 2023). This protocol was approved by the University of California, San Francisco Institutional Review Board (protocol 16-20442), and all patients provided written consent. In this protocol, patients were administered local anesthesia with 2% lidocaine, and right heart catheterization was performed using a 6 French introducer sheath (St. Jude Medical, St. Paul, Minnesota, USA) and 6 French ballon-tipped pulmonary artery wedge catheter (Teleflex, Morrisville, North Carolina, USA). PCWP was measured with the right heart catheter (RHC) using standard RHC protocol; further details about this measurement process can be found in Shandhi et al. (2022). Cardiac output was obtained using thermodilution, and it was normalized to cardiac index (CI) using the patient’s estimated body surface area. After baseline measurements, a subset of patients were administered vasodilators, either a sublingual spray of nitroglycerin (400 or 800 mcg) or an intravenous infusion of nitroprusside (0.3 mcg/kg/min, titrated by 0.4 mcg/kg/min every 5 minutes until hemodynamic effect was achieved). When the administering physician determined peak hemodynamic effect occurred, PCWP and CI were remeasured. Subject-wise hemodynamic parameters are provided in **Supplementary Table 1**. Throughout the protocol, continuous non-invasive signals were collected using a custom-made wearable patch placed on the mid-sternum. This device collects ECG sampled at 1 kHz using a single lead and adhesive-backed Ag/AgCl electrodes and triaxial SCG (dorso-ventral, head-to-foot, and lateral axes) sampled at 500 Hz using an accelerometer. All signals in the dataset were resampled to 500 Hz.

For this dataset, decompensated hypervolemia was defined as PCWP greater or equal to 18 mmHg and CI less than or equal to 2.2 L/min/m^2^; PCWP less than 18 mmHg or CI greater than 2.2 L/min/m^2^ was considered compensated hypervolemia (Forrester et al., 1977). Of the 28 patients who underwent vasodilation, 12 were decompensated at baseline and compensated after vasodilation. One subject was excluded due to poor ECG quality, resulting in 11 subjects evaluated (seven male and four female, 30 to 66 years old, 55.8 to 130.8 kg). For use in our analysis, we extracted 60 seconds of data during the baseline PCWP measurement (decompensation) and 60 seconds of data during the vasodilation PCWP measurement (compensation).

### 2.2 Hypovolemia datasets

This investigation combined two different studies of porcine models undergoing exsanguination to induce hypovolemia. In the first dataset (Zia et al., 2020b; Kimball et al., 2021; Cho et al., 2025, Cho et al., 2026), six Yorkshire swine (three castrated male and three female, 51.5 to 71.4 kg) were anesthetized using xylazine (0.5 mg/kg) and telazol (4 mg/kg), maintained with inhaled isoflurane (up to 5%), and placed on mechanical ventilation. Baseline blood volume was estimated using the Evans blue dye method (Ertl et al., 2007). Arterial pressure was recorded invasively from the right carotid artery with a fluid-filled catheter inserted through a vascular introducer. Hypovolemia was induced by passive blood removal through an arterial line in increments of 7% total blood volume. After each removal, exsanguination was paused for 5 to 10 minutes to allow vitals to stabilize or completely stopped if cardiovascular collapse occurred. Cardiovascular collapse was defined in this procedure by a 20% drop in mean arterial pressure (MAP) from baseline. Throughout the protocol, continuous non-invasive signals were recorded with a sampling frequency of 2 kHz using the BIOPAC MP160 acquisition system (BIOPAC Systems Inc., Goleta, California, USA). ECG signals were collected in a three-lead Einthoven Lead II configuration using adhesive-backed Ag/AgCl electrodes interfacing with the BIOPAC ECG 100C amplifier. Triaxial SCG signals were collected from the mid-sternum using the ADXL354 accelerometer (Analog Devices Inc., Norwood, Massachusetts, USA) interfacing with the BIOPAC HLT100C transducer interface module. Following the experimental procedure, the swine were euthanized by either injection of potassium chloride (1–2 mEq/kg) or exsanguination while still under anesthesia. This protocol was approved by the Institutional Animal Care and Use Committees of the Georgia Institute of Technology (protocol A100276) and Translational Testing and Training Labs Inc. (protocol GT48P) and the Department of the Navy Bureau of Medicine and Surgery.

In the second dataset (Rezaei et al., 2026; Tangolar et al., 2026), nine Yorkshire swine (all female, 42 to 50 kg) were anesthetized using xylazine (5 mg/kg) and ketamine (25 mg/kg), maintained with inhaled isoflurane (1.5-4%), and placed on mechanical ventilation. Baseline blood volume was estimated as 65 ml/kg of body weight. Arterial pressure was recorded invasively from the abdominal artery with a transducer-tipped catheter. Hypovolemia was induced by passive blood removal through an arterial line in increments of 5% total blood volume. After each removal, exsanguination was paused for 10 minutes to allow vitals to stabilize or completely stopped if cardiovascular collapse occurred. Cardiovascular collapse was defined in this procedure by a sustained 20 mmHg reduction in MAP from baseline. Throughout this protocol, continuous non-invasive signals were recorded with a sampling frequency of 512 Hz using the FDA-cleared Cardiotag device (Cardiosense, Inc., Chicago, Illinois, USA) placed on the mid-sternum. This device collects ECG using a single lead and adhesive-backed Ag/AgCl electrodes and triaxial SCG using an accelerometer. Following the experimental procedure, the swine were euthanized by exsanguination while still under anesthesia. This protocol was approved by the Institutional Animal Care and Use Committee of the University of Maryland School of Medicine (protocol AUP-00000093) and the Department of the Navy Bureau of Medicine and Surgery Veterinary Affairs Office.

To unify the two porcine studies, compensated hypovolemia was defined as the waiting period after the first blood removal, corresponding to 7% and 5% of total blood volume removed for the first and second datasets, respectively. Decompensated hypovolemia was defined as the first waiting or stopping period when a 20% reduction in MAP from baseline occurred. Combining the two datasets, we evaluated 15 swine undergoing exsanguination to induce hypovolemia. Subject-wise hemodynamic parameters are provided in **Supplementary Table 2**. To match the available data from the hypervolemia dataset, ECG and SCG signals were down sampled to 500 Hz, and a single 60 second window extracted from the middle of these 5 to 10-minute stabilization periods were extracted for use in analysis. The middle section of these periods was selected to ensure that the hemodynamic effects observed were due to the presence or lack of blood, not the removal act, and that minimal motion artifacts from the experimenters starting or stopping the procedure were corrupting the signal.

### 2.3 Signal processing

ECG and SCG signals were filtered using linear-phase finite impulse response bandpass filters with a Kaiser window with cutoff frequencies of 1 to 40 Hz and 2 to 39 Hz, respectively. After filtering, R-peaks were extracted from the ECG signal, visually inspected, and manually corrected if an incorrect time point was selected. The hypervolemia dataset was further visually inspected for arrythmias such as premature ventricular contractions and premature atrial contractions; if an arrhythmia was observed, data proceeding the previous clean R-peak to the next clean R-peak was removed. We wanted to evaluate variability only due to hemodynamic conditions, not electrophysiology. Nevertheless, in future studies the presence of arrhythmia may be included as well in algorithms to classify compensatory status, to augment hemodynamic content investigated here. The cleaned R-peak to R-peak intervals, also known as Normal-to-Normal (NN) intervals, were then used to segment the SCG signals into heartbeats. The reason for this approach was that the variability from volume status would be confounded by variability from arrhythmia if such ectopic beats were included in the analysis. The goal of the analysis was to understand the sources of variability specifically related to volume status, rather than to differences in preload resulting from a premature beat and the associated shortened diastolic filling time, and/or differences in ventricular pumping function associated with electrical conduction abnormalities present in premature ventricular contractions in particular.

**Figure 2:**
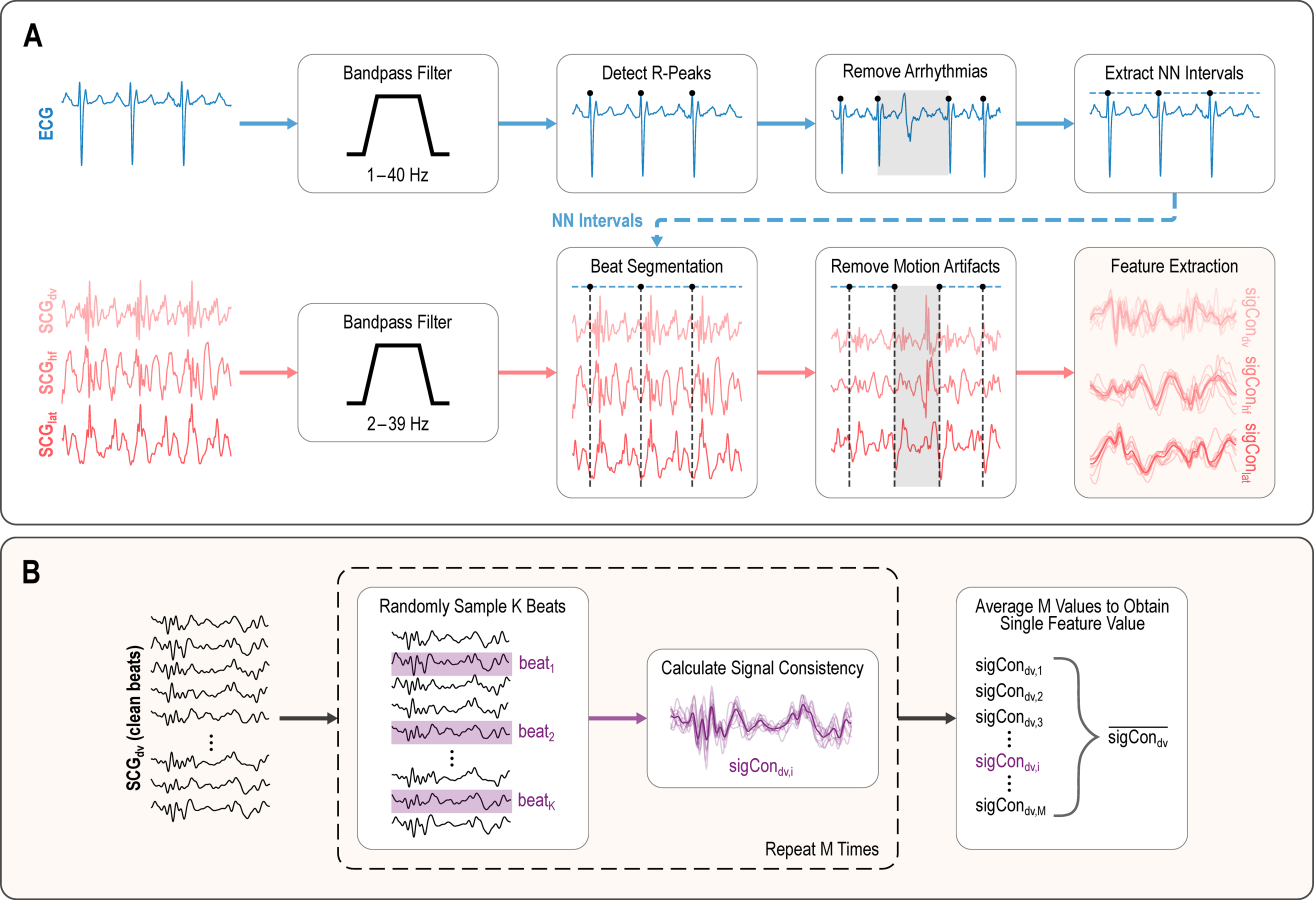
**(A)** ECG and triaxial SCG signal processing pipeline for a single subject and 60 second severity level (compensation or decompensation) window. **(B)** details of signal consistency feature extraction from part **(A)**.

SCG beats containing motion artifacts, either due to subject movement or researcher interference, were removed through a manual process guided by signal quality indexing (SQI). Each beat was assigned an SQI value based on the method proposed by Gazi et al. (2021), where an SQI value closer to 1 indicates greater similarity to the Woody’s algorithm-based template of beats within a 30 second window preceding the evaluated beat. The 30 seconds preceding the extracted 1-minute window were used for the purposes of SQI calculation but were not included in signal consistency evaluation. Each SCG axis was processed separately and then beats in each axis were aligned. Beats that had an amplitude greater than 4 standard deviations from the mean amplitude or had an SQI less than 0.2 were flagged for visual inspection. We examined these flagged beats and the beats around them and manually removed any beats corrupted by motion artifacts. A manual selection process was used to ensure only beats corresponding to motion artifacts were removed, as current automatic signal quality-based beat exclusion processes may remove beats simply affected by physiological state. Specifically, we removed beats with large fluctuating amplitudes that did not occur during events typically associated with aortic valve opening or closing in the SCG signal; if a beat was found to contain a motion artifact in at least one axis, it was removed from the other axes as well. The overall signal processing procedure is visualized in **Figure 2A**. All signal processing was completed in MATLAB 2025a (The MathWorks, Inc., Natick, MA, USA).

### 2.4 Signal consistency feature

Once the clean SCG beats were obtained, beat-to-beat variability was quantified for the compensated and decompensated periods. Inan et al. (2010) previously developed a technique to estimate the noise-to-signal ratio (NSR) of cardiogenic vibration heartbeats (ballistocardiogram signals). While this method was used initially to determine the noise level in the signal, after removing heartbeats corrupted by motion and other sources of noise the metric actually allows for the direct quantification of variability in the morphology of heartbeats compared to the ensemble average. Pandia (2017) adapted this method to create the signal consistency metric, which is found by creating a template from a group of beats to estimate the underlying cardiac signal, comparing each beat to this template to determine an “inconsistency” metric for each beat, and calculating the “consistency” of the group as the inverse of the average of their “inconsistency” metrics. While many other methods could be used to define consistency of heartbeat morphology within a recording, we decided to use this simple and interpretable method for signal consistency such that the results from the study could readily be understood and appreciated for future studies.

#### 2.4.1 Feature extraction

To calculate a single signal consistency feature value for each severity level, compensation and decompensation, K=10 random beats were sampled and the individual signal consistency metric, described below, was computed. This was repeated a total of M=150 times, ensuring each 10-beat combination was not already used, and all signal consistency metric values were averaged to obtain a single feature value for the severity level. This process is visualized in **Figure 2B**.

The random sampling method was chosen because some subjects retain consecutive beats after poor-quality beats are removed while others do not, and thus it is not possible to find sequential heartbeats without arrhythmia, motion artifact, or low signal quality. A purely temporal analysis that relies only on consecutive samples may introduce inconsistencies across subjects and act as a confounding factor, as consecutive beats are more likely to be consistent with one another and artificially inflate the averaged signal consistency measure if random sampling is not used.

A repetition count of 150 was selected to represent a large sample size to ensure that the average signal consistency value was representative of the true signal consistency and not influenced by small amounts of motion artifact noise that may not have been removed from individual beats. The selection of M = 150 was confirmed by calculating signal consistency 100 times for each subject, blood volume condition, and SCG axis using a different random seed, producing different sets of random beats, each time. We performed this process from M = 10 to M = 500 in steps of 10 and calculated the standard deviation of signal consistency values for each M. In all cases, the standard deviation leveled out, and the “elbows” of each of these curves, as calculated by the geometric triangle method, were all below M = 150, as visualized in **Supplementary Figures 1, 2**. The elbows of the curves were examined in order to balance minimal deviation for recalculations between signal consistency and minimal computational load.

#### 2.4.2 Signal consistency metric calculation

The following equations detail how to calculate the signal consistency metric for the *m*th group of *M* groups of SCG beats. The *m*th group of beats is defined as a vector, *X*, of *K* beats cropped to the shortest beat length of *T* time point samples, where


$$X=[\begin{array}{cc} \begin{array}{cc} {\vec{x}}_{1} & {\vec{x}}_{2} \end{array} & \begin{array}{cc} \cdots & {\vec{x}}_{K} \end{array} \end{array}]$$

and each individual beat, ${\vec{x}}_{k}$, is


$${\vec{x}}_{k}=[\begin{array}{c} \begin{array}{c} x_{k}[1]\\ x_{k}[2] \end{array}\\ \begin{array}{c} \vdots \\ x_{k}[T] \end{array} \end{array}]\;.$$

The underlying cardiac signal template for this group of beats, $\vec{s}$, is defined as the ensemble average of all *K* beats,


$$\vec{s}=\frac{1}{K}\sum_{k=1}^{K}{\vec{x}}_{k}\;,$$

where


$$\vec{s}=[\begin{array}{c} \begin{array}{c} s[1]\\ s[2] \end{array}\\ \begin{array}{c} \vdots \\ s[T] \end{array} \end{array}].$$

This template is scaled for each *k*th beat using a constant amplitude factor, $a_{k}$. Amplitude scaling is performed because we want to specifically examine waveform morphology variation, not the amplitude changes in SCG that occur due to respiration. The constant amplitude factor is defined as a vector of all *K* beats,


$$\vec{a}=\;[\begin{array}{cc} \begin{array}{cc} a_{1} & a_{2} \end{array} & \begin{array}{cc} \cdots & a_{K} \end{array} \end{array}],$$

and calculated by multiplying the pseudoinverse of $\vec{s}$ by *X*,


$$\vec{a}={\left({\vec{s}}^{⊤}\vec{s}\right)}^{-1}{\vec{s}}^{⊤}X\;.$$

The difference, ${\vec{v}}_{k}$, of the scaled template from the *k*th beat is defined as


$$V=X-\vec{s}\vec{a}\;,$$

where


$$V=[\begin{array}{cc} \begin{array}{cc} {\vec{v}}_{1} & {\vec{v}}_{2} \end{array} & \begin{array}{cc} \cdots & {\vec{v}}_{K} \end{array} \end{array}]$$

and


$${\vec{v}}_{k}=[\begin{array}{c} \begin{array}{c} v_{k}[1]\\ v_{k}[2] \end{array}\\ \begin{array}{c} \vdots \\ v_{k}[T] \end{array} \end{array}]\;.$$

The normalized variance of each *k*th beat, ${\hat{var}}_{k}$, is then calculated by dividing the variance of the beat’s difference by the variance of the template beat


$${\hat{var}}_{k}=\frac{\sigma_{{\vec{v}}_{k}}^{2}}{\sigma_{\vec{s}}^{2}}\;.$$

Finally, the signal consistency metric for the *m*th group of *K* beats is calculated as


$$sigCon_{m}=\frac{K}{\sum_{k=1}^{K}{\hat{var}}_{k}}\;,$$

and the single signal consistency feature value, obtained from all M groups of beats, is calculated as th


$$\bar{sigCon}=\frac{1}{M}\sum_{m=1}^{M}sigCon_{m}\;.$$

### 2.5 Statistical analysis

We evaluated the hypervolemia and hypovolemia populations separately to evaluate changes in signal consistency. We were specifically interested in whether there was a significant change between compensation versus decompensation measurable in any or all SCG axes. Since we had both compensation and decompensation measurements for each subject, a paired statistical test was used to assess statistically significant differences. Due to outliers present in both the hypervolemia and hypovolemia populations, we used the non-parametric Wilcoxon signed-rank test. The non-parametric Mann-Whitney U test was used to assess differences in hypervolemia subjects administered nitroglycerin versus nitroprusside for vasodilation. A threshold of α = 0.05 was set to determine significance. All statistical analyses were completed in MATLAB 2025a (The MathWorks, Inc., Natick, MA, USA).

## 3 Results

**Figure 3:**
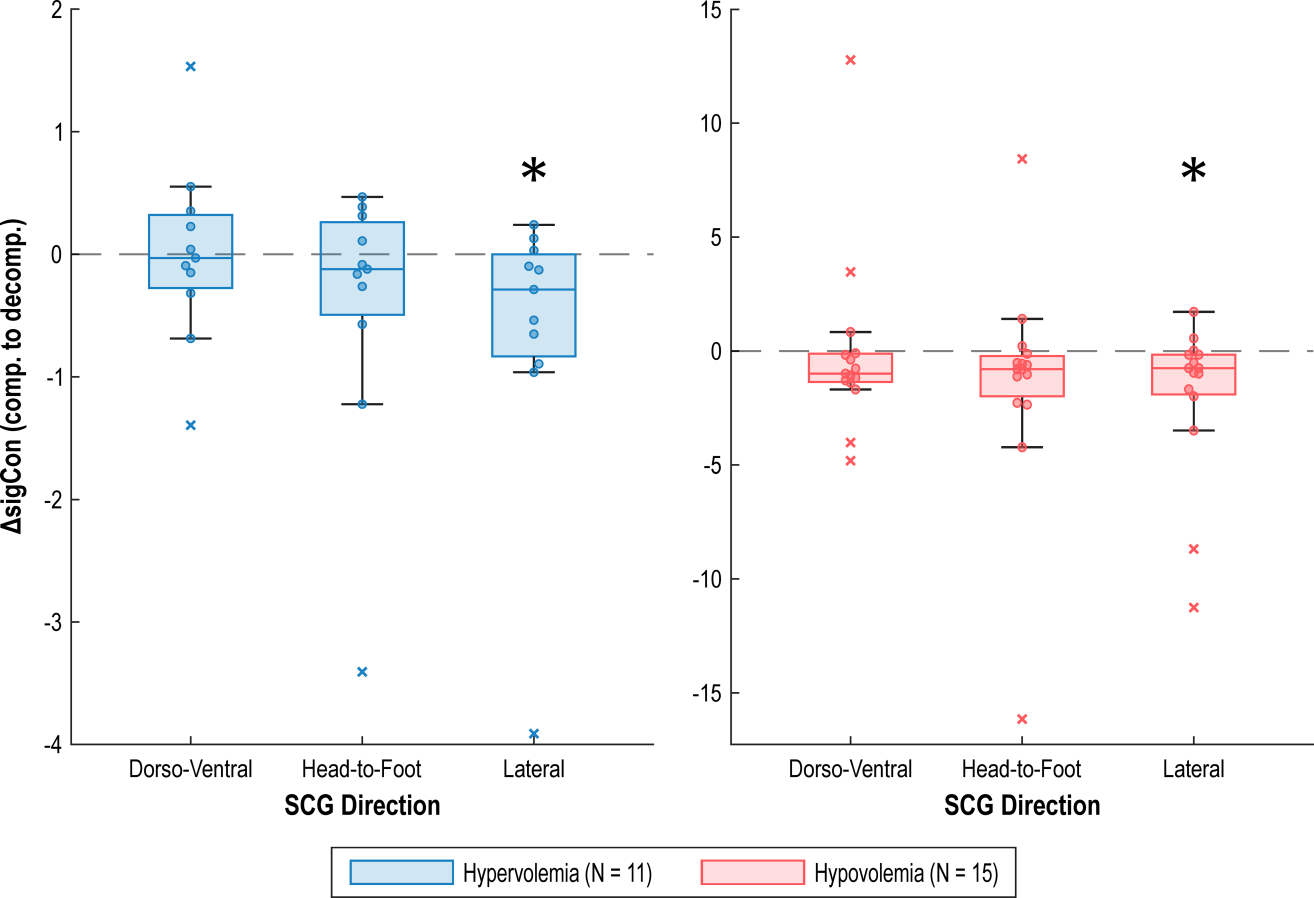
Box-and-whisker plots of change in signal consistency from compensation to decompensation for the three SCG axes, with each subject represented by a single data point. Statistical significance (p < 0.05) calculated by the Wilcoxon signed-rank test is denoted by “*”.

**Figure 3** visualizes the results across all three SCG axes, and numerical details are provided in **Tables 1**, **2**; these findings are presented in the following subsections. Signal consistency scores for each subject at compensation and decompensation are provided in **Supplementary Tables 4** through **6**. While the vasodilators given to induce compensated hypervolemia produce two different effects – nitroglycerin (4 subjects) primarily reduces preload and nitroprusside (7 subjects) reduces both preload and afterload (Yancy et al., 2013) – we did not observe a significant difference in signal consistency between the two groups for any axis (**Supplementary Table 7**), so their results are reported together. More subjects are required to examine a difference in signal consistency between the two vasodilators.

**Table 1:** Metrics detailing change in signal consistency from compensation to decompensation for the hypervolemic subject group (N = 11); statistical significance (p < 0.05) calculated by the Wilcoxon signed-rank test is denoted by “*”.

SCG axis	Median	Interquartile range	Wilcoxon signed-rank test results		
			p-value	Test statistic (W)	Effect size (r)
*Dorso-ventral*	-0.030	-0.276 to 0.320	1.000	33	0.013
*Head-to-foot*	-0.121	-0.493 to 0.262	0.413	23	-0.247
Lateral*	-0.288	-0.833 to -0.001	0.042*	10	-0.613

**Table 2:** Metrics detailing change in signal consistency from compensation to decompensation for the hypovolemic subject group (N = 15); statistical significance (p < 0.05) calculated by the Wilcoxon signed-rank test is denoted by “*”.

SCG axis	Median	Interquartile range	Wilcoxon signed-rank test results		
			p-value	Test statistic (W)	Effect size (r)
*Dorso-ventral*	-0.992	-1.360 to -0.113	0.121	32	-0.401
*Head-to-foot*	-0.794	-1.983 to -0.221	0.055	26	-0.495
Lateral*	-0.751	-1.907 to -0.158	0.012*	17	-0.645

### 3.1 SCG dorso-ventral and head-to-foot axes

We did not observe a statistically significant change in signal consistency of the SCG dorso-ventral axis when comparing compensation to decompensation in hypervolemia (p = 1.000) nor hypovolemia (p = 0.121). Additionally, we did not observe a statistically significant change in signal consistency of the SCG head-to-foot axis for hypervolemia (p = 0.413) nor hypovolemia (p = 0.055).

### 3.2 SCG lateral axis

**Figure 4:**
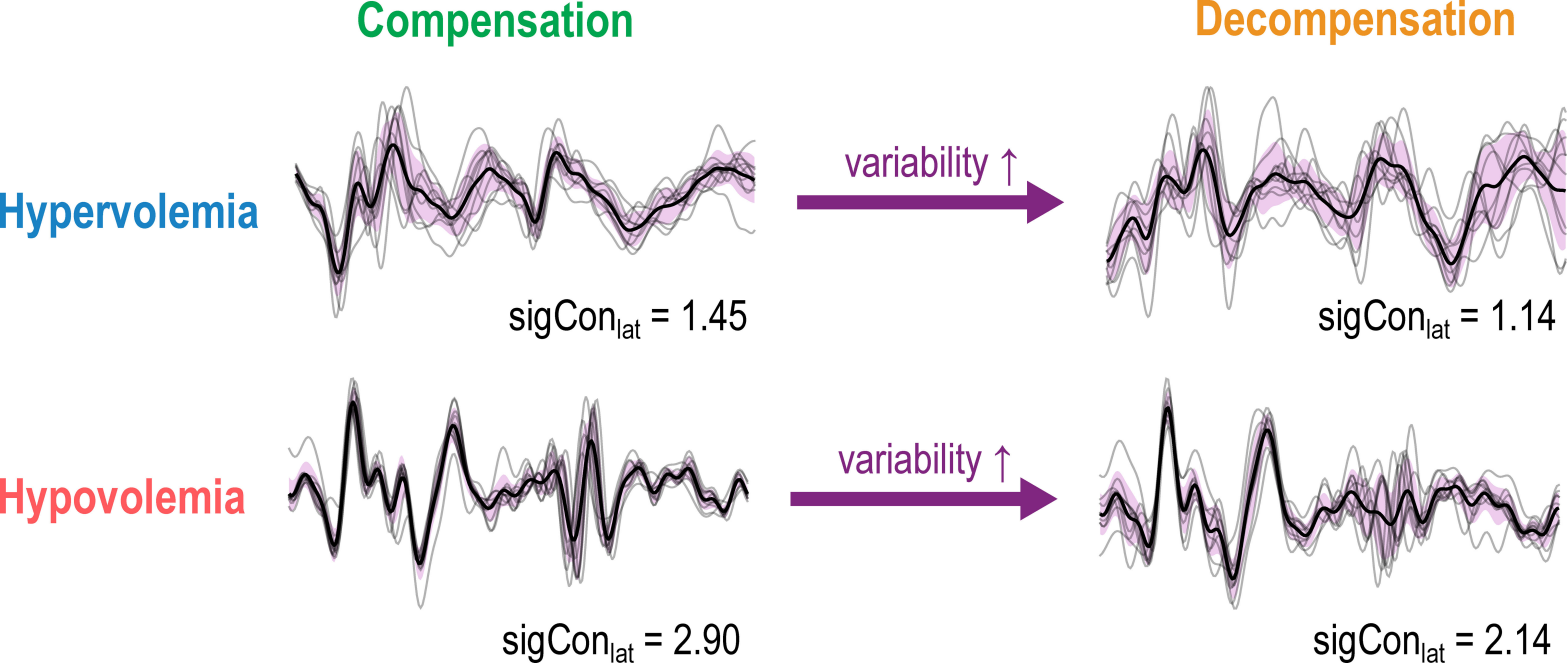
Examples of single signal consistency values from the lateral SCG axis for a hypervolemia and a hypovolemia subject prior to averaging, representative of the populations’ decrease in average signal consistency – and therefore increase in variability – from compensation to decompensation. Thin black lines represent 10 individual beats, and a thick black line represents the ensemble average used to evaluate signal consistency. The standard deviation of the 10 beats, although it is not used in signal consistency calculation, has been highlighted in purple to emphasize differences in variability.

We observed a statistically significant decrease in signal consistency of the SCG lateral axis from both compensated to decompensated hypervolemia (p = 0.042) and hypovolemia (p = 0.012). Representative changes in the lateral SCG signal are represented in **Figure 4**.

## 4 Discussion

### 4.1 Principal finding

In this study, we found that higher variability of the lateral SCG axis is associated with decompensation while lower variability is associated with compensation for both hypervolemia and hypovolemia. These results imply that the SCG lateral axis provides physiologically meaningful information about the volume status of the heart. This is the first study to our knowledge that examines the SCG signal – particularly the head-to-foot and lateral axes – in the context of both extreme suboptimal volume status conditions, hypervolemia and hypovolemia, and from compensation to decompensation. Furthermore, we believe SCG signal consistency is one of the first physiological metrics, beyond HRV, found to deflect in the same direction for worsening states of both hypervolemia and hypovolemia.

### 4.2 Novel examination of SCG morphology variability and cardiac function

Measuring heartbeat-wise physiological variability is not a new concept; however, commonly used variability metrics are limited, with little focus on full-waveform fluctuations. HRV, quantifying beat-to-beat changes in heart rate and most often measured via ECG, is one of the most prominent metrics due to ease of measurement and understanding; it has been applied in countless scenarios from investigating cognitive and emotional control to quantifying cardiovascular risk (Pham et al., 2021). Other variability metrics include arterial pressure or echocardiography-based stroke volume variation and PPG-based pulse pressure variation, both used as predictors of fluid responsiveness (Berkenstadt et al., 2001; Bednarczyk et al., 2017). However, these metrics only quantify how the timing or amplitude of a single fiducial point, defined by a distinctive event, changes from beat to beat, greatly simplifying physiological changes. Variability metrics utilizing the full beat waveform have been shown to be additionally informative towards the assessment of abnormal cardiovascular function. T-wave alternans, beat-to-beat variability of the ECG T-wave, has been linked to susceptibility to ventricular arrhythmias (Narayan, 2006), and whole-beat to beat variability metrics have been used with ECG and photoplethysmogram signals to identify acute coronary syndrome patients at high risk of death (Liu et al., 2014) and predict short-term surgical outcomes (Ho et al., 2024), respectively.

For the SCG signal, variability is still most often assessed through changes in the timing of extracted features such as the aortic opening and closing points on the SCG beat. These can be used to assess fluctuations in pre-ejection period and left ventricular ejection time (Korzeniowska-Kubacka et al., 2006; Di Rienzo et al., 2013; Nawar et al., 2026) but provide little to no information about other cardiomechanical changes that may occur from beat to beat. In contrast, beat-wise variability of the SCG signal, especially the quantification of full-beat morphological changes, is rarely explored as a metric. Similarity measures have been used to detect individual beats (Centracchio et al., 2023), assess signal quality (Zia et al., 2020a), and analyze the effects of respiration (Azad et al., 2019; Gamage et al., 2020; Centracchio et al., 2025), but we believe this is one of the first works to study SCG waveform similarity as a physiological metric indicative of health status. These results indicate that the variability of SCG morphology is an important feature that changes with blood volume status, and it should be examined in other clinical conditions as well.

### 4.3 Lateral SCG variability as a biomarker of sub-optimal blood volume status

A majority of studies investigating SCG focus mainly on the dorso-ventral axis (Inan et al., 2015), likely due to its prominent fiducial points that have been associated with the pumping function of the heart such as aortic valve opening and closing. However, this study highlights the importance of the lateral axis and how it may provide additional cardiovascular information such as blood volume and flow. These results are supported by Erin and Semiz (2024), who showed that spectral features derived from the lateral SCG axis are important in the classification of stenosis versus regurgitation, which are heart valve diseases that affect blood flow; Shandhi et al. (2022) also found that morphology changes in the lateral SCG axis from baseline to vasodilation are important in estimating changes in PCWP and pulmonary artery pressure, related to pulmonary congestion, of heart failure patients. The latter finding is particularly relevant since the filling characteristics of the heart are disrupted in sub-optimal volume status conditions, and thus the importance of the lateral SCG axis for characterizing variability associated with sub-optimal volume status may be attributed to the disruption in filling of the heart (i.e., sub-optimal preload). Our study makes a noteworthy observation that in both the hypervolemic and hypovolemic cases, the lateral SCG variability significantly deflected in the same direction, demonstrating its promise as a biomarker of suboptimal blood volume status.

### 4.4 Potential insight into compensatory-related physiological changes

Decreasing HRV, associated with increased sympathetic nervous system activation (“fight-or-flight”), is observed with both worsening hypervolemia and hypovolemia (De Jong and Randall, 2005; Cooke et al., 2008; Ferrario et al., 2014). However, seemingly contradictory, we have observed increased SCG morphological variability from compensated to decompensated sub-optimal blood volume conditions in this study. The exact physiological mechanisms behind this change are unknown but can be hypothesized with current understanding of cardiovascular mechanisms.

Effects of respiration on the vascular system occur regardless of compensation status; inspiration and expiration cause fluctuations in intrathoracic pressure, which is observed in both spontaneous breathing and positive pressure ventilated individuals. These fluctuations in intrathoracic pressures subsequently cause respiration-mediated fluctuations in the heart’s preload and, to a lesser extent, afterload (Brecher and Hubay, 1955; Berger and Takala, 2018; Jozwiak and Teboul, 2024). At the same time, the stroke volume of the heart is modulated by preload, or the stretch of myocardial fibers; this relationship is characterized by the Frank-Starling Mechanism. Stroke volume increases with preload, graphically described by a steep ascending limb, until the muscle approaches its stretching limit, described by a plateau and potentially descending limb (Frank, 1895; Starling, 1918). Researchers have already found that in healthy humans, SCG morphology is altered along the respiration cycle (Azad et al., 2019; Gamage et al., 2020).

With hypovolemia, individuals begin higher on the ascending limb of the Starling curve and move down this limb as they lose blood, as preload is reduced. At lower portions of the limb, small changes in preload caused by respiration results in larger changes in stroke volume. This is a commonly observed phenomenon, and techniques measuring the respiration-based variability of stroke volume, such as pulse pressure variability, are commonly used to assess fluid responsiveness (Marik, 2009). In terms of our results, higher SCG morphology variability during decompensated hypovolemia may capture this higher variability in the heart’s contractional force.

In contrast, the physiological mechanisms captured by our signal consistency metric for hypervolemic patients may be more complicated. There are some claims that after cardiac muscles are stretched to their limit, further increasing afterload could cause decreases in stroke volume (MacGregor et al., 1974; Ross, 1983); with afterload varying with respiration, perhaps we are capturing variations in stroke volume through our SCG signal consistency metric during decompensated hypervolemia. Another potential source of increased variability, particularly in decompensated heart failure patients, is the decoupling of left ventricular end diastolic pressure (LVEDP), often measured by PCWP and used as a surrogate for preload, and left ventricular end diastolic volume. Left ventricular filling has been shown to be constrained by pericardial and right ventricular pressures, which are normally low but increased in congestive heart failure and volume overload; thus, when blood volume is significantly increased, preload and subsequently stroke volume decrease even though LVEDP is increased (Moore et al., 2001). Since pressures in the heart are modulated by respiration-based changes in intrathoracic pressure, we may be capturing preload-mediated variations in stroke volume during decompensated hypervolemia, but fewer variations during compensation due to reduced pressure and influence of the pericardium and right ventricle.

In addition to respiration-dependent changes in stroke volume, perhaps changes in respiration itself contribute to increased SCG variability at decompensation. Irregular respiration has been associated with worsening states of hypervolemia and hypovolemia: shortness of breath (dyspnea) is a common symptom of decompensated heart failure and fluid overload (Schiff et al., 2003; Yancy et al., 2013), and hyperventilation is associated with worsening hypovolemia (Convertino et al., 2021). We do not expect respiration to affect our specific hypovolemia results, as throughout the course of each procedure, the respiratory rate and tidal volume of the ventilator for each pig were kept relatively stable. However, if decompensation- dependent alterations in respiration are present in the studied hypervolemic patients, these are likely to have impact on SCG morphology, as studies such as Gamage et al. (2020); Skoric et al. (2022), and Centracchio et al. (2025) have already demonstrated that respiration phase and depth modulate SCG morphology. Overall, further investigation is required to determine the exact mechanisms behind increased SCG morphology variability at decompensation for both hypervolemia and hypovolemia.

### 4.5 Limitations

The analyses in this paper were limited to small sample sizes; future research should include larger study populations to validate results. For hypervolemia, we only investigated patients experiencing heart failure, but patients experiencing other hypervolemia-related conditions such as kidney failure should be looked at in future studies. Similarly, instead of a human hypovolemia model, we examined a porcine model, as it is not ethical to hemorrhage large amounts of blood from humans. While methods of inducing hypovolemia in humans such as lower body negative pressure exist, they can only simulate early compensatory stages of hypovolemia (Convertino et al., 2023); thus, only animal models can be used for the controlled study of decompensated hypovolemia. In the future, the animal should be observed non-anesthetized and ambulatory to better mimic the conditions of traumatic hypovolemia. Finally, for this study heartbeats containing arrhythmias or large motion artifacts were removed manually, but in future work an automatic process would guarantee consistency and allow for larger scale or real-time analysis.

### 4.6 Conclusions and future work

Currently, this research has demonstrated a novel connection between compensation status and SCG morphology variability, particularly in the lateral SCG axis. These results indicate that, while currently underutilized, the lateral SCG axis shares an important relation to blood volume, and the mechanisms behind this should be explored. Further research should be conducted to determine which aspects of SCG morphology change from compensation to decompensation, with particular focus on cardio-pulmonary interactions, as this will provide a better understanding of the SCG signal and how it relates to heart functionality.

In this research, compensated and decompensated hypervolemia and hypovolemia were studied as binary conditions for the initial investigation of SCG signal consistency as a metric of decompensation status. With the current findings that signal consistency decreases from compensation to decompensation, future work should investigate the non-binary progression from healthy to various levels of compensation to decompensation. This may include investigating SCG variability in a longitudinal study of the progression of heart failure and as blood is progressively exsanguinated from a porcine model. Furthermore, as we have already connected a decrease in signal consistency to worsening absolute hypovolemia, this relation should also be studied in the context of relative hypovolemia.

In the long-term, the signal consistency feature investigated in this research and the supplemental discovery of additional variability-based features – from SCG or other signals – could allow for the creation of more accurate baseline-dependent or novel baseline-independent classification or regression models of compensated to decompensated sub-optimal volume status. These models, combined with an inexpensive non-invasive device, would provide actionable information in a multitude of settings such as combat casualty care, pre-hospital emergency care evaluation, at-home monitoring of various diseases, and monitoring of fluid status in extreme environments, allowing remote, preventative assessment of these conditions.

## Data Availability

The raw data supporting the conclusions of this article will be made available by the authors, without undue reservation.
