# Supplementary material for: Quantifying changes in triaxial seismocardiography variability due to sub-optimal volume status
Source: Front Physiol. 2026 May 18;17:1823345. doi: 10.3389/fphys.2026.1823345 (PMC13222946; doi:10.3389/fphys.2026.1823345)
Supplement: Supplementary file 1 [file DataSheet1.docx]

Supplementary Material

# Supplementary Figures and Tables

## Supplementary Figures

**Supplementary Figure 1.** Analysis of choice of M (random beat sampling repetition count) on signal consistency reproducibility for hypervolemic subjects. Each color represents a subject. Solid curves represent standard deviation of signal consistency computed over 100 iterations. Dotted lines represent the corresponding “elbow” of each curve. Dotted lines for all colors/subjects are not visible due to overlap. M = 150 was chosen as an optimal balance between reproducibility and computational efficiency.


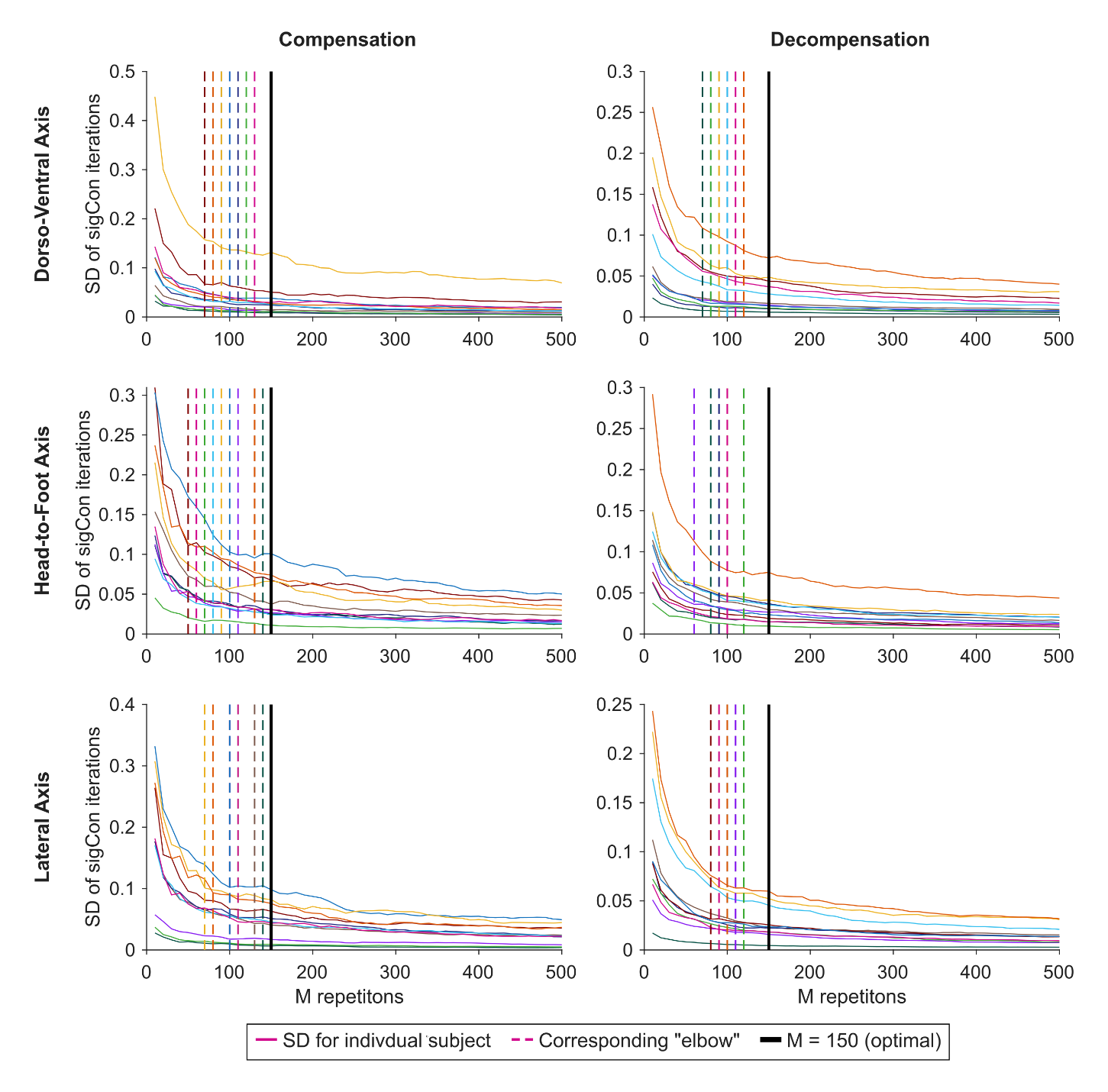


**Supplementary Figure 2.** Analysis of choice of M (random beat sampling repetition count) on signal consistency reproducibility for hypovolemic subjects. Each color represents a subject. Solid curves represent standard deviation of signal consistency computed over 100 iterations. Dotted lines represent the corresponding “elbow” of each curve. Dotted lines for all colors/subjects are not visible due to overlap. M = 150 was chosen as an optimal balance between reproducibility and computational efficiency.


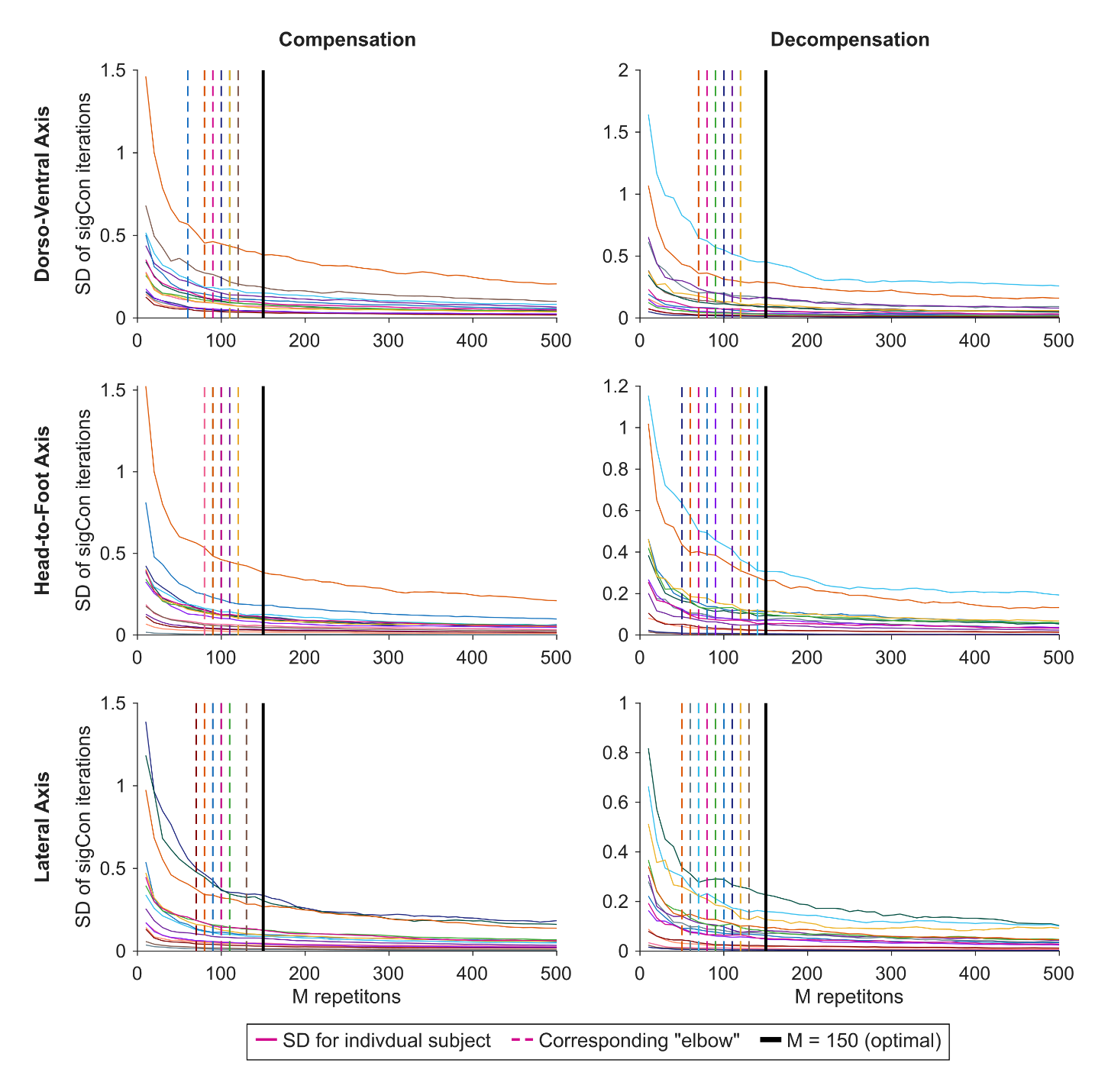


## Supplementary Tables

**Supplementary Table 1.** Hemodynamic details collected from individual hypervolemic (heart failure) patients during compensation and decompensation. Values are averaged from the entire pulmonary capillary wedge pressure procedure, which contains the 60 second period examined in this study.

| **Sub. ID** | **Vasodilator** | **Severity Level** | **Heart Rate (beats/min)** | **Pulmonary Capillary Wedge Pressure (mmHg)** | **Cardiac Index (L/min/m^2^)** | **Stroke Volume (mL/beat)** |
| --- | --- | --- | --- | --- | --- | --- |
| 1 | Nitroprusside | comp. | 72 | 10 | 3.09 | 104.1 |
|  |  | decomp. | 82 | 35 | 1.65 | 43.1 |
| 2 | Nitroprusside | comp. | 96 | 12 | 2.38 | 45.0 |
|  |  | decomp. | 101 | 31 | 1.81 | 35.9 |
| 3 | Nitroglycerin | comp. | 70 | 16 | 1.22 | 46.9 |
|  |  | decomp. | 41 | 22 | 1.32 | 53.9 |
| 4 | Nitroprusside | comp. | 79 | 5 | 1.88 | 40.1 |
|  |  | decomp. | 70 | 18 | 1.77 | 42.1 |
| 5 | Nitroglycerin | comp. | 67 | 13 | 2.23 | 81.1 |
|  |  | decomp. | 70 | 18 | 1.90 | 89.8 |
| 6 | Nitroglycerin | comp. | 91 | 16 | 2.06 | 37.2 |
|  |  | decomp. | 93 | 22 | 2.00 | 37.3 |
| 7 | Nitroprusside | comp. | 88 | 12 | 2.75 | 65.9 |
|  |  | decomp. | 80 | 24 | 2.00 | 42.6 |
| 8 | Nitroprusside | comp. | 88 | 13 | 3.60 | 83.5 |
|  |  | decomp. | 72 | 26 | 1.74 | 58.6 |
| 9 | Nitroprusside | comp. | 82 | 10 | 2.71 | 55.5 |
|  |  | decomp. | 95 | 18 | 2.05 | 37.9 |
| 10 | Nitroprusside | comp. | 88 | 14 | 2.63 | 69.1 |
|  |  | decomp. | 79 | 24 | 2.04 | 51.7 |
| 11 | Nitroglycerin | comp. | 103 | 15 | 2.96 | 65.3 |
|  |  | decomp. | 100 | 26 | 2.09 | 46.6 |

**Supplementary Table 2.** Hemodynamic details collected from individual hypovolemic subjects during compensation and decompensation. “T3L” refers to the first hypovolemia dataset described in the text; “UMD” refers to the second. Values for the T3L subjects are averaged from approximately the last minute of the stabilization periods. Values for the UMD subjects are averaged from the middle 60% of the stabilization periods. Differences in sampling periods are due to differing protocols.

| **Sub. ID** | **Dataset** | **Severity Level** | **Heart Rate (beats/ min)** | **Mean Arterial Pressure (mmHg)** | **Cardiac Output (L/min)** | **Stroke Volume (mL/beat)** | **Total Peripheral Resistance (mmHg* min/L)** |
| --- | --- | --- | --- | --- | --- | --- | --- |
| 12 | T3L | comp. | 113 | 46 | 39.8 | 4.50 | 10.2 |
|  |  | decomp. | 126 | 42 | 34.4 | 4.33 | 9.7 |
| 13 | T3L | comp. | 80 | 56 | 71.3 | 5.70 | 9.8 |
|  |  | decomp. | 113 | 35 | 28.5 | 3.22 | 10.9 |
| 14 | T3L | comp. | 69 | 56 | 56.5 | 3.90 | 14.4 |
|  |  | decomp. | 64 | 42 | 50.3 | 3.22 | 13.0 |
| 15 | T3L | comp. | 61 | 63 | 70.0 | 4.27 | 14.8 |
|  |  | decomp. | 55 | 38 | 57.6 | 3.17 | 12.0 |
| 16 | T3L | comp. | 93 | 48 | 51.8 | 4.82 | 10.0 |
|  |  | decomp. | 133 | 42 | 33.5 | 4.46 | 9.4 |
| 17 | T3L | comp. | 94 | 56 | 57.8 | 5.43 | 10.3 |
|  |  | decomp. | 120 | 43 | 34.6 | 4.15 | 10.4 |
| 18 | UMD | comp. | 132.1 | 58.0 | 23.0 | 3.04 | 19.1 |
|  |  | decomp. | 126.7 | 41.7 | 16.6 | 2.11 | 19.8 |
| 19 | UMD | comp. | 126.7 | 56.9 | 22.1 | 2.80 | 20.3 |
|  |  | decomp. | 133.6 | 44.3 | 16.2 | 2.16 | 20.5 |
| 20 | UMD | comp. | 146.0 | 58.2 | 28.4 | 4.14 | 14.1 |
|  |  | decomp. | 175.5 | 47.4 | 14.1 | 2.47 | 19.2 |
| 21 | UMD | comp. | 134.3 | 43.2 | 21.8 | 2.93 | 14.8 |
|  |  | decomp. | 136.7 | 30.1 | 12.2 | 1.67 | 18.0 |
| 22 | UMD | comp. | 122.6 | 35.8 | 10.5 | 1.29 | 27.7 |
|  |  | decomp. | 121.7 | 27.4 | 8.3 | 1.01 | 27.1 |
| 23 | UMD | comp. | 110.4 | 59.7 | 27.4 | 3.02 | 19.8 |
|  |  | decomp. | 111.8 | 42.0 | 15.3 | 1.72 | 24.5 |
| 24 | UMD | comp. | 129.7 | 59.0 | 22.4 | 2.90 | 20.3 |
|  |  | decomp. | 130.8 | 44.3 | 15.5 | 2.03 | 21.8 |
| 25 | UMD | comp. | 138.9 | 48.4 | 12.7 | 1.76 | 27.4 |
|  |  | decomp. | 140.4 | 38.0 | 9.7 | 1.36 | 28.0 |
| 26 | UMD | comp. | 96.9 | 55.6 | 23.3 | 2.26 | 24.6 |
|  |  | decomp. | 124.9 | 40.4 | 14.8 | 1.85 | 21.8 |

**Supplementary Table 3.** Heartbeat exclusion details for individual subjects. Signal quality index is abbreviated as SQI.

| **Sub. ID** | **Blood Volume Condition** | **Severity Level** | **Initial # Beats within 60 sec** | **# Beats Excluded** | | | | **Total # Beats Remaining** | **SQI of Remaining Beats** | | | | | |
| --- | --- | --- | --- | --- | --- | --- | --- | --- | --- | --- | --- | --- | --- | --- |
|  |  |  |  | **… Due to Arrhythmias** | **… Due to Motion Artifacts/ Other Noise Sources** | | **Total** |  |  |  |  |  |  |  |
|  |  |  |  |  | **Identified with High Amp. Flag** | **Identified with Low SQI Flag** |  |  | **DV axis mean** | **DV axis s.d.** | **HF axis mean** | **HF axis s.d.** | **LAT axis mean** | **LAT axis s.d.** |
| 1 | hyper | comp. | 77 | 30 | 0 | 0 | 30 | 47 | **0.23** | 0.06 | **0.41** | 0.14 | **0.34** | 0.14 |
|  |  | decomp. | 84 | 0 | 4 | 7 | 11 | 73 | **0.22** | 0.06 | **0.44** | 0.10 | **0.36** | 0.08 |
| 2 | hyper | comp. | 95 | 8 | 0 | 3 | 11 | 84 | **0.26** | 0.07 | **0.32** | 0.10 | **0.37** | 0.13 |
|  |  | decomp. | 86 | 7 | 2 | 3 | 12 | 74 | **0.14** | 0.03 | **0.32** | 0.09 | **0.21** | 0.09 |
| 3 | hyper | comp. | 72 | 13 | 0 | 2 | 15 | 57 | **0.17** | 0.05 | **0.22** | 0.10 | **0.17** | 0.05 |
|  |  | decomp. | 74 | 28 | 0 | 0 | 28 | 46 | **0.17** | 0.05 | **0.21** | 0.08 | **0.15** | 0.04 |
| 4 | hyper | comp. | 79 | 0 | 6 | 7 | 13 | 66 | **0.40** | 0.07 | **0.45** | 0.11 | **0.42** | 0.08 |
|  |  | decomp. | 69 | 0 | 10 | 0 | 10 | 59 | **0.41** | 0.04 | **0.37** | 0.07 | **0.45** | 0.07 |
| 5 | hyper | comp. | 65 | 8 | 0 | 0 | 8 | 57 | **0.26** | 0.04 | **0.36** | 0.09 | **0.25** | 0.05 |
|  |  | decomp. | 65 | 8 | 0 | 0 | 8 | 57 | **0.27** | 0.06 | **0.33** | 0.11 | **0.29** | 0.07 |
| 6 | hyper | comp. | 84 | 26 | 2 | 1 | 29 | 55 | **0.29** | 0.05 | **0.23** | 0.08 | **0.32** | 0.07 |
|  |  | decomp. | 87 | 40 | 0 | 0 | 40 | 47 | **0.26** | 0.06 | **0.24** | 0.06 | **0.34** | 0.09 |
| 7 | hyper | comp. | 89 | 8 | 0 | 0 | 8 | 81 | **0.43** | 0.07 | **0.52** | 0.09 | **0.59** | 0.10 |
|  |  | decomp. | 94 | 2 | 2 | 1 | 5 | 89 | **0.24** | 0.06 | **0.29** | 0.09 | **0.37** | 0.10 |
| 8 | hyper | comp. | 86 | 6 | 9 | 22 | 37 | 49 | **0.18** | 0.04 | **0.19** | 0.07 | **0.19** | 0.05 |
|  |  | decomp. | 87 | 15 | 4 | 16 | 35 | 52 | **0.15** | 0.03 | **0.14** | 0.06 | **0.18** | 0.08 |
| 9 | hyper | comp. | 90 | 0 | 3 | 0 | 3 | 87 | **0.44** | 0.04 | **0.45** | 0.05 | **0.51** | 0.07 |
|  |  | decomp. | 96 | 0 | 1 | 2 | 3 | 93 | **0.33** | 0.04 | **0.39** | 0.08 | **0.45** | 0.08 |
| 10 | hyper | comp. | 90 | 0 | 1 | 0 | 1 | 89 | **0.30** | 0.06 | **0.44** | 0.10 | **0.54** | 0.11 |
|  |  | decomp. | 82 | 0 | 0 | 0 | 0 | 82 | **0.36** | 0.08 | **0.49** | 0.12 | **0.48** | 0.12 |
| 11 | hyper | comp. | 104 | 0 | 21 | 4 | 25 | 79 | **0.38** | 0.05 | **0.32** | 0.07 | **0.36** | 0.10 |
|  |  | decomp. | 99 | 0 | 2 | 61 | 63 | 36 | **0.30** | 0.05 | **0.22** | 0.06 | **0.23** | 0.06 |
| 12 | hypo | comp. | 87 | 0 | 4 | 2 | 6 | 81 | **0.29** | 0.04 | **0.16** | 0.02 | **0.20** | 0.04 |
|  |  | decomp. | 126 | 0 | 0 | 0 | 0 | 126 | **0.40** | 0.02 | **0.35** | 0.07 | **0.34** | 0.06 |
| 13 | hypo | comp. | 81 | 0 | 0 | 0 | 0 | 81 | **0.30** | 0.04 | **0.31** | 0.05 | **0.37** | 0.08 |
|  |  | decomp. | 105 | 0 | 8 | 11 | 19 | 86 | **0.21** | 0.04 | **0.20** | 0.04 | **0.24** | 0.08 |
| 14 | hypo | comp. | 66 | 0 | 10 | 0 | 10 | 56 | **0.42** | 0.02 | **0.41** | 0.07 | **0.49** | 0.06 |
|  |  | decomp. | 63 | 0 | 1 | 0 | 1 | 62 | **0.44** | 0.02 | **0.41** | 0.08 | **0.51** | 0.07 |
| 15 | hypo | comp. | 60 | 0 | 0 | 0 | 0 | 60 | **0.38** | 0.04 | **0.43** | 0.06 | **0.46** | 0.10 |
|  |  | decomp. | 56 | 0 | 0 | 0 | 0 | 56 | **0.33** | 0.04 | **0.24** | 0.03 | **0.32** | 0.04 |
| 16 | hypo | comp. | 96 | 0 | 4 | 0 | 4 | 92 | **0.35** | 0.03 | **0.22** | 0.03 | **0.20** | 0.03 |
|  |  | decomp. | 127 | 0 | 9 | 1 | 10 | 117 | **0.32** | 0.03 | **0.11** | 0.02 | **0.14** | 0.02 |
| 17 | hypo | comp. | 94 | 0 | 15 | 1 | 16 | 76 | **0.22** | 0.04 | **0.18** | 0.03 | **0.16** | 0.02 |
|  |  | decomp. | 125 | 0 | 6 | 9 | 15 | 110 | **0.17** | 0.04 | **0.13** | 0.03 | **0.12** | 0.02 |
| 18 | hypo | comp. | 130 | 0 | 0 | 0 | 0 | 130 | **0.41** | 0.08 | **0.36** | 0.09 | **0.62** | 0.05 |
|  |  | decomp. | 123 | 0 | 3 | 0 | 3 | 120 | **0.43** | 0.07 | **0.42** | 0.11 | **0.46** | 0.12 |
| 19 | hypo | comp. | 125 | 0 | 0 | 0 | 0 | 125 | **0.20** | 0.05 | **0.23** | 0.04 | **0.24** | 0.04 |
|  |  | decomp. | 132 | 0 | 0 | 0 | 0 | 132 | **0.20** | 0.03 | **0.25** | 0.06 | **0.21** | 0.04 |
| 20 | hypo | comp. | 144 | 0 | 0 | 0 | 0 | 144 | **0.31** | 0.05 | **0.27** | 0.03 | **0.28** | 0.03 |
|  |  | decomp. | 176 | 0 | 0 | 0 | 0 | 176 | **0.28** | 0.06 | **0.27** | 0.03 | **0.25** | 0.02 |
| 21 | hypo | comp. | 134 | 0 | 0 | 0 | 0 | 134 | **0.52** | 0.02 | **0.44** | 0.05 | **0.41** | 0.07 |
|  |  | decomp. | 137 | 0 | 0 | 0 | 0 | 137 | **0.55** | 0.03 | **0.49** | 0.04 | **0.43** | 0.07 |
| 22 | hypo | comp. | 121 | 0 | 0 | 0 | 0 | 121 | **0.34** | 0.05 | **0.49** | 0.06 | **0.38** | 0.06 |
|  |  | decomp. | 120 | 0 | 2 | 0 | 2 | 118 | **0.31** | 0.04 | **0.43** | 0.08 | **0.33** | 0.07 |
| 23 | hypo | comp. | 109 | 0 | 0 | 0 | 0 | 109 | **0.34** | 0.07 | **0.34** | 0.07 | **0.44** | 0.09 |
|  |  | decomp. | 111 | 0 | 1 | 1 | 2 | 109 | **0.32** | 0.06 | **0.39** | 0.09 | **0.42** | 0.10 |
| 24 | hypo | comp. | 128 | 0 | 0 | 0 | 0 | 128 | **0.35** | 0.06 | **0.50** | 0.05 | **0.48** | 0.10 |
|  |  | decomp. | 130 | 0 | 0 | 0 | 0 | 130 | **0.41** | 0.06 | **0.47** | 0.05 | **0.50** | 0.09 |
| 25 | hypo | comp. | 138 | 0 | 0 | 0 | 0 | 138 | **0.50** | 0.02 | **0.57** | 0.03 | **0.47** | 0.03 |
|  |  | decomp. | 139 | 0 | 0 | 0 | 0 | 139 | **0.52** | 0.04 | **0.50** | 0.08 | **0.34** | 0.09 |
| 26 | hypo | comp. | 92 | 0 | 0 | 0 | 0 | 92 | **0.43** | 0.05 | **0.38** | 0.03 | **0.39** | 0.04 |
|  |  | decomp. | 122 | 0 | 0 | 0 | 0 | 122 | **0.35** | 0.09 | **0.20** | 0.05 | **0.21** | 0.04 |

**Supplementary Table 4.** Disaggregated signal consistency scores calculated with the dorso-ventral SCG axis.

| **Subject ID** | **Blood Volume Condition** | **Compensation** | **Decompensation** | **Change from Comp. to Decomp.** |
| --- | --- | --- | --- | --- |
| 1 | hyper | 0.620 | 0.590 | -0.030 |
| 2 | hyper | 1.034 | 0.348 | -0.686 |
| 3 | hyper | 0.365 | 0.272 | -0.093 |
| 4 | hyper | 2.518 | 3.070 | 0.552 |
| 5 | hyper | 0.818 | 0.668 | -0.150 |
| 6 | hyper | 1.205 | 1.557 | 0.352 |
| 7 | hyper | 1.966 | 0.572 | -1.394 |
| 8 | hyper | 0.528 | 0.568 | 0.040 |
| 9 | hyper | 2.800 | 2.482 | -0.318 |
| 10 | hyper | 1.278 | 2.810 | 1.532 |
| 11 | hyper | 2.092 | 2.319 | 0.227 |
| 12 | hypo | 1.481 | 4.952 | 3.470 |
| 13 | hypo | 2.163 | 0.991 | -1.172 |
| 14 | hypo | 7.877 | 7.782 | -0.094 |
| 15 | hypo | 2.861 | 1.176 | -1.685 |
| 16 | hypo | 8.724 | 3.908 | -4.816 |
| 17 | hypo | 0.690 | 0.523 | -0.167 |
| 18 | hypo | 3.837 | 2.846 | -0.992 |
| 19 | hypo | 0.919 | 0.550 | -0.369 |
| 20 | hypo | 2.239 | 1.170 | -1.069 |
| 21 | hypo | 6.345 | 19.115 | 12.771 |
| 22 | hypo | 3.515 | 2.228 | -1.287 |
| 23 | hypo | 2.012 | 1.241 | -0.771 |
| 24 | hypo | 3.247 | 4.083 | 0.836 |
| 25 | hypo | 11.423 | 10.039 | -1.384 |
| 26 | hypo | 5.387 | 1.371 | -4.016 |

**Supplementary Table 5.** Disaggregated signal consistency scores, calculated with the head-to-foot SCG axis, for hypervolemic subjects.

| **Subject ID** | **Blood Volume Condition** | **Compensation** | **Decompensation** | **Change from Comp. to Decomp.** |
| --- | --- | --- | --- | --- |
| 1 | hyper | 1.421 | 1.530 | 0.109 |
| 2 | hyper | 1.056 | 1.368 | 0.313 |
| 3 | hyper | 0.707 | 0.544 | -0.163 |
| 4 | hyper | 2.476 | 1.253 | -1.223 |
| 5 | hyper | 1.165 | 0.902 | -0.263 |
| 6 | hyper | 0.825 | 1.212 | 0.387 |
| 7 | hyper | 4.183 | 0.777 | -3.406 |
| 8 | hyper | 0.421 | 0.337 | -0.085 |
| 9 | hyper | 2.682 | 2.561 | -0.121 |
| 10 | hyper | 2.640 | 3.108 | 0.468 |
| 11 | hyper | 1.253 | 0.682 | -0.570 |
| 12 | hypo | 0.176 | 1.589 | 1.413 |
| 13 | hypo | 1.075 | 0.552 | -0.523 |
| 14 | hypo | 1.195 | 1.408 | 0.214 |
| 15 | hypo | 2.453 | 0.181 | -2.272 |
| 16 | hypo | 1.283 | 0.168 | -1.115 |
| 17 | hypo | 0.735 | 0.178 | -0.557 |
| 18 | hypo | 3.240 | 2.446 | -0.794 |
| 19 | hypo | 0.898 | 0.778 | -0.121 |
| 20 | hypo | 2.962 | 2.161 | -0.801 |
| 21 | hypo | 5.322 | 13.754 | 8.433 |
| 22 | hypo | 5.889 | 3.535 | -2.354 |
| 23 | hypo | 4.036 | 3.010 | -1.026 |
| 24 | hypo | 7.314 | 6.700 | -0.614 |
| 25 | hypo | 24.945 | 8.798 | -16.147 |
| 26 | hypo | 5.556 | 1.333 | -4.223 |

**Supplementary Table 6.** Disaggregated signal consistency scores, calculated with the lateral SCG axis, for hypervolemic subjects.

| **Subject ID** | **Blood Volume Condition** | **Compensation** | **Decompensation** | **Change from Comp. to Decomp.** |
| --- | --- | --- | --- | --- |
| 1 | hyper | 1.440 | 1.153 | -0.288 |
| 2 | hyper | 1.591 | 0.697 | -0.894 |
| 3 | hyper | 0.310 | 0.212 | -0.098 |
| 4 | hyper | 1.870 | 1.902 | 0.032 |
| 5 | hyper | 0.809 | 0.682 | -0.128 |
| 6 | hyper | 1.234 | 1.364 | 0.130 |
| 7 | hyper | 5.042 | 1.132 | -3.910 |
| 8 | hyper | 0.346 | 0.586 | 0.240 |
| 9 | hyper | 4.339 | 3.802 | -0.537 |
| 10 | hyper | 3.600 | 2.638 | -0.962 |
| 11 | hyper | 1.352 | 0.702 | -0.650 |
| 12 | hypo | 0.343 | 2.066 | 1.723 |
| 13 | hypo | 1.462 | 0.511 | -0.952 |
| 14 | hypo | 1.667 | 1.689 | 0.022 |
| 15 | hypo | 1.504 | 0.516 | -0.988 |
| 16 | hypo | 0.756 | 0.244 | -0.512 |
| 17 | hypo | 2.146 | 0.163 | -1.983 |
| 18 | hypo | 12.535 | 3.850 | -8.685 |
| 19 | hypo | 0.950 | 0.791 | -0.158 |
| 20 | hypo | 2.880 | 2.131 | -0.749 |
| 21 | hypo | 4.295 | 4.857 | 0.562 |
| 22 | hypo | 3.517 | 1.840 | -1.677 |
| 23 | hypo | 3.629 | 3.471 | -0.158 |
| 24 | hypo | 6.705 | 5.954 | -0.751 |
| 25 | hypo | 13.832 | 2.575 | -11.257 |
| 26 | hypo | 5.169 | 1.682 | -3.487 |

**Supplementary Table 7.** Comparison of hypervolemic patients receiving nitroglycerin versus nitroprusside as a vasodilator.

| **SCG axis** | **Change in sigCon, group median (IQR)** | | **Mann-Whitney U test results** | | |
| --- | --- | --- | --- | --- | --- |
|  | **Nitroprusside** (n = 7) | **Nitroglycerin** (n = 4) | **p-value** | **U statistic** | **Effect size (r)** |
| DV | -0.030 (-0.594 to 0.424) | 0.067 (-0.122 to 0.289) | 0.788 | 12 | 0.085 |
| HF | -0.085 (-0.948 to 0.262) | -0.213 (-0.417 to 0.112) | 0.788 | 16 | 0.085 |
| LAT | -0.537 (-0.945 to -0.048) | -0.110 (-0.389 to 0.016) | 0.412 | 9 | 0.256 |
